# Genetic Diversity and *Wolbachia* (Rickettsiales: Anaplasmataceae) Prevalence Within a Remnant Population of Regal Fritillary, *Argynnis idalia* (Lepidoptera: Nymphalidae), in South-Central Pennsylvania

**DOI:** 10.1093/jisesa/ieac006

**Published:** 2022-02-16

**Authors:** Ilga Rutins, Sarah Schannauer, Sharil Orellana, Harrison Laukhuff, Eric Lang, Timothy Becker, Erika McKinney, Kayli Thomas, Virginia Tilden, Mark Swartz, Jaime E Blair

**Affiliations:** 1 Department of Biology, Franklin & Marshall College, Lancaster, PA 17603, USA; 2 ZooAmerica North American Wildlife Park, Hershey, PA 17033, USA; 3 The Pennsylvania Department of Military and Veterans Affairs, Fort Indiantown Gap National Guard Training Center, Annville, PA 17003, USA

**Keywords:** butterfly, disease monitoring, conservation, microsatellite, mitochondrial DNA

## Abstract

Eastern populations of the North American regal fritillary, *Argynnis idalia* Drury (1773), have been largely extirpated over the past half century. Here we report on the last remaining population of eastern regal fritillaries, located within a military installation in south-central Pennsylvania. Samples were obtained from field specimens during two years of annual monitoring, and from females collected for captive rearing over a five year period. Nuclear microsatellite and mitochondrial sequence data do not suggest subdivision within this population, but excess nuclear homozygosity indicates negative impacts on genetic diversity likely due to small population size and potential inbreeding effects. Molecular assays did not detect *Wolbachia* endosymbionts in field specimens of regal fritillary, but sympatric *Argynnis* sister species showed high prevalence of *Wolbachia* infected individuals. Our results inform ongoing conservation and reintroduction projects, designed to protect the last remaining regal fritillary population from extirpation in the eastern United States.

Butterfly populations worldwide face a variety of complex threats, including habitat fragmentation, environmental degradation, and many other anthropogenic impacts (Potts et al. 2010, Wilson and Maclean 2011, Wagner 2020). Climate change specifically plays a role in altering species distributions and trophic interactions, often in unpredictable ways, e.g., between butterflies and their food resources (Schweiger et al. 2008), or through exposure to new or emerging diseases and pathogens (Gallana et al. 2013). Small, isolated populations often then experience a reduction in genetic diversity due to inbreeding and reduced gene flow with other subpopulations (Saccheri et al. 1998, Mattila et al. 2012). Effective conservation measures therefore must consider both local and landscape level environmental conditions as well as the genetic viability of isolated populations in order to mitigate the threat of local extinctions.

The North American regal fritillary, *Argynnis* (syn. *Speyeria*) *idalia*, is a large, univoltine species that historically inhabited mixed and tall-grass prairies across the Midwest, Great Plains, and Northeast of the United States (Selby 2007). Habitat loss primarily due to agricultural conversion has restricted most Midwestern populations, and Northeastern populations have been extirpated over the past 50 years likely due to development and succession in grasslands (Williams 2002, Selby 2007, Sims 2017). The eastern regal fritillary, *A. idalia idalia*, proposed as a separate subspecies (Williams 2001), is currently found only on a collection of small meadows housed within a military training facility in south-central Pennsylvania, the Fort Indiantown Gap National Guard Training Center (FIG-NGTC). A similarly isolated remnant population at a military installation in Virginia has since become extirpated, with individuals last observed in the late 1990s (Chazel et al. 2010). Conservation efforts over the past twenty years have carefully examined habitat requirements for the eastern regal (Swartz et al. 2015), as well as population size (Ferster and Vulinec 2010, Zografou et al. 2017), genetic diversity (Williams 2002, Williams et al. 2003, Keyghobadi et al. 2006, 2013), and phenological changes (Zografou et al. 2021). Intensive management methods, such as prescribed burns and removal of invasive species, have been employed to maintain appropriate habitat for eastern regals and their food resources, particularly violets (*Viola* sp.) which are crucial for larval development (Latham et al. 2007, Adamidis et al. 2019). Over the past decade, a captive rearing program has been initiated for the eastern regal; the goal of this program is to produce quantities of individuals that can be used to establish self-sustaining populations at appropriate reintroduction sites within Pennsylvania (Becker 2016).

While environmental degradation plays a substantial role in the decline of butterfly populations, pathogens and parasites can also impact survival and persistence, especially for small populations with low genetic diversity. Endosymbiotic *Wolbachia* bacteria are of particular concern for Lepidoptera and other insects, as infections can interfere with the physiology and reproductive success of the host (Werren et al. 2008, Correa and Ballard 2016). Modelling suggests that greater than 65% of insect and up to 80% of Lepidopteran species are infected with *Wolbachia*, although prevalence within a species is likely to vary (Hilgenboecker et al. 2008, Ahmed et al. 2015). As reproductive parasites that are vertically transmitted via host eggs, *Wolbachia* infections can skew sex ratios through feminization or killing of male offspring, and reduce overall reproductive output through cytoplasmic incompatibility between infected males and uninfected females (Werren et al. 2008). Over 575 unique *Wolbachia* strain types have been described to date based on multilocus sequence typing of five housekeeping genes (Baldo et al. 2006, Jolley et al. 2018); of these, strain type 41 (ST-41) has been reported as the most common *Wolbachia* strain associated with Lepidopterans worldwide, although many strains have been documented from diverse hosts (Ahmed et al. 2016, Ilinsky and Kosterin 2017). Diagnosing *Wolbachia* infections and monitoring strain types are therefore important considerations for conservation strategies, especially those that include captive breeding, rearing, or translocation of individuals among subpopulations (Hamm et al. 2014).

In this study, our goals were to revisit estimates of genetic diversity within the eastern regal fritillary population at FIG-NGTC, and to examine *Wolbachia* prevalence and strain type within the resident *Argynnis* species. Because the Pennsylvania regal fritillary population is considered critically imperiled (Schweitzer 2021), nonlethal sampling approaches were used in the field to obtain tissue for molecular analyses. We utilized existing nuclear microsatellite (Williams et al. 2002) and mitochondrial (Williams 2002, Keyghobadi et al. 2006) markers previously developed for *A. idalia* to measure diversity and potential population subdivision. We also modified existing molecular assays to detect *Wolbachia* DNA in our *Argynnis* samples; *Wolbachia* bacteria have been detected in a variety of somatic tissues in insects, including wings and legs (Pietri et al. 2016), which are common targets for non-lethal sampling in butterflies (Koscinski et al. 2011). Our results suggest that the eastern regal fritillary population at FIG-NGTC likely suffers negative genetic impacts from inbreeding, but no significant subdivision among meadows. In addition, while the eastern regal fritillaries do not appear to harbor *Wolbachia* infections, sympatric *Argynnis* species are infected, at varying prevalence. Our results suggest that continued monitoring of genetic diversity and *Wolbachia* infection status, especially for those *A. idalia* females collected for captive rearing, is critical for conservation strategies moving forward.

## Methods

### Specimen Collection and DNA Extraction

Each field season, eastern regal fritillary subpopulations at FIG-NGTC are surveyed for abundance via Pollard-walk methodologies (Pollard and Yates 1993); comprehensive mark-recapture surveys are also implemented every 4–5 years to assess population size and other parameters (MS, unpublished data). Tissue samples were collected during annual population monitoring in July 2017 and in July & August 2019 using a nonlethal method similar to previous studies (Starks and Peters 2002). A single hindleg was removed at approximately the trochanter from each *A. idalia* individual using clean forceps and placed in a sterile microcentrifuge tube or glass vial. In 2019, hindleg samples were also collected from sympatric great spangled (*A. cybele* Fabricius, 1775) and aphrodite (*A. aphrodite* Fabricius, 1787) fritillaries for comparative analysis. Samples were transported to the lab at ambient temperature, and frozen at −20°C prior to processing. DNA was extracted from each sample using the DNeasy Blood & Tissue Kit (Qiagen, Germantown, MD) following the manufacturer's protocol with modification. Individual hindleg samples were cut into approx. 5–10 mm segments using a sterile razor blade, and incubated overnight at 56°C in Buffer ATL and proteinase K. DNA was eluted with two washes of 25 µl sterile ultrapure water (50 µl total) and stored at −20°C.

Tissue samples were also obtained from gravid *A. idalia* females collected at FIG-NGTC in mid to late August (2016–2020) for captive rearing. Females were maintained in the lab through ovipositing or until a pre-designated egg quota was reached; the thousands of eggs produced by these individuals were reared through the caterpillar stage and used for reintroduction efforts or additional rearing research. DNA was extracted from abdomen samples or from individual hindlegs as described above. Unhatched eggs and dead or diseased larvae and caterpillars were stored at −20°C for later analysis.

### *Wolbachia* Detection and Strain Typing

A multiplex PCR-based assay was developed to detect the presence of *Wolbachia* bacterial DNA coextracted from infected host samples. Arthropod-specific 28S rDNA primers obtained from the literature and optimized for Lepidoptera were used: 28SF3633 (5′-TAC CGT GAG GGA AAG TTG AAA-3′; Choudhury and Werren 2006 as cited in Nice et al. 2009) and 28SbLEP (5′-CG GAC GGA ACC AGC TAC TA-3′; modified from 28Sb, Whiting et al. 1997). The amplification of a 800–900 bp fragment served as an internal control, indicating a successful DNA extraction and limited presence of PCR inhibitors. Primers W-SpecF (5′-CAT ACC TAT TCG AAG GGA TAG-3′) and W-SpecR (5′-AGC TTC GAG TGA AAC CAA TTC-3′) were used to amplify a region of the *Wolbachia* 16S rDNA (Werren and Windsor 2000); the presence of a 438 bp fragment in addition to the larger insect 28S amplicon suggests the presence of *Wolbachia* DNA within the sample, and thus a possible infection. A common lab strain of *Drosophila melanogaster* infected with *Wolbachia* (clonal complex ST-13) was used as a positive control for all assays. PCR reactions contained 1X standard *Taq* reaction buffer (New England Biolabs, Ipswich, MA) with additional MgCl_2_ to total 2.5 mM, 200 µM dNTPs, 0.2 µM each forward and reverse primer, 1 U *Taq* polymerase, 1 µl template DNA (approx. 10 ng), and were brought to 20 µl total volume with sterile ultrapure water. The thermal cycler conditions were as follows: 94°C for 2 min; 35 cycles of 94°C for 30 s, 60°C for 45 s, 72°C for 60 s; final extension at 72°C for 10 min. Assays were scored by visualizing the amplicon products on a 1% agarose gel. Representative 28S amplicons for the three *Argynnis* species examined here were sequenced and deposited in GenBank (accession numbers MZ079856-MZ079858).

To identify false positives from the multiplex assay, all samples were also evaluated with *Wolbachia*-specific primers for fructose-bisphosphate aldolase (*fbpA*) as suggested by previous studies (Simões et al. 2011, Hamm et al. 2014). Primer sequences for *fbpA* were obtained from the *Wolbachia* PubMLST database (Baldo et al. 2006, Jolley et al. 2018); PCR reactions contained 1X *Taq* Master Mix (New England Biolabs), 0.8 µM forward and reverse primer, 1 µl template DNA, and were brought to 20 µl total volume with sterile ultrapure water. The thermal cycler conditions were as follows: 94°C for 2 min; 35 cycles of 94°C for 30 s, 59°C for 45 s, 72°C for 90 s; final extension at 72°C for 10 min. Amplicons were visualized on a 1% agarose gel. For those samples with a positive result from the multiplex assay and the *fbpA* assay, the remaining four *Wolbachia* MLST loci (*coxA*, *ftsZ*, *gatB*, *hcpA*; [Baldo et al. 2006]) were amplified as described above except with an annealing temperature of 54°C for the thermal cycler protocol (primer concentrations for *hcpA* were 0.4 µM). The *Wolbachia* outer surface protein (*wsp*) marker was also amplified to complement the MLST loci (Baldo et al. 2006); PCR conditions were the same as those for *fbpA* described above. All amplicons were cleaned with the enzymatic Exo-CIP Rapid PCR Cleanup kit (New England Biolabs), and sent to the University of Kentucky HealthCare Genomics Core Laboratory for Sanger sequencing. Raw trace files were edited using Sequencher version 5.4 (Gene Codes Corporation, Ann Arbor, MI) to remove primer sequences and generate consensus sequences from forward and reverse reads. Consensus sequences were then used to query the *Wolbachia* PubMLST database to determine the allelic profile and strain type for each positive *Argynnis* sample.

### Microsatellite Diversity Analysis

Four polymorphic nuclear microsatellite loci previously developed for *Argynnis idalia* (Williams et al. 2002) were utilized here to examine genetic diversity within the FIG-NGTC population. Fluorescently-labeled forward primers were custom ordered (Applied Biosystems, Foster City, CA), each with a different reporter dye on the 5′ end (Si13: 6-FAM, Si17: PET, Si18: NED, Si31: VIC). Multiplex PCR reactions contained 1.2X Phusion High-Fidelity PCR Master Mix (New England Biolabs), 0.2 µM each forward and reverse primer, 1 µl template DNA, and were brought to 15 µl total volume with sterile ultrapure water. The thermal cycler conditions were as follows: 98°C for 1 min; 28 cycles of 98°C for 15 s, 57°C for 30 s, 72°C for 30 s; final extension at 72°C for 30 min. Labeled amplicons were sent to GENEWIZ (South Plainfield, NJ) for ABI3730xl capillary-based fragment analysis with a LIZ-500 size standard. Raw data files were processed in Geneious Prime version 2020.2.5 (https://www.geneious.com/); predicted peaks were adjusted manually as needed, particularly for stutter bands, and binning was based on a 2-bp repeat unit (4-bp for Si17) with size ranges as reported previously (Williams et al. 2002). The total number of alleles per locus, observed (*H*_*O*_) and expected (*H*_*E*_) heterozygosities, departure from Hardy-Weinberg equilibrium, and measurements of population differentiation (*F*_*ST*_, *G*_*ST*_) were calculated using GeneAlEx v.6.5 (Peakall and Smouse 2012) and PopGenReport v.1.6.6 (Adamack and Gruber 2014); allelic richness and the frequency of null alleles for each locus were also estimated in PopGenReport.

### Mitochondrial Sequence Variation and Phylogenetic Analysis

Sequences were generated from representative samples of the three *Argynnis* species studied here for two mitochondrial loci: partial cytochrome oxidase subunits I and II (COI+II) and partial NADH dehydrogenase subunit 4 (ND4). For the COI+II locus, primers C1-J-2183 (5′-CAA CAT TTA TTT TGA TTT TTT GG-3′) and TK-N-3772 (5′-GAC CAT TAC TTG CTT TCA GTC ATC T-3′) were used (Williams 2002); PCR reactions contained 1X standard *Taq* reaction buffer (New England Biolabs) with additional MgCl_2_ to total 3.5 mM, 200 µM dNTPs, 0.2 µM forward and reverse primer, 1 U *Taq* polymerase, 1 µl template DNA, and were brought to 20 µl total volume with sterile ultrapure water. The thermal cycler conditions were as follows: 94°C for 2 min; 35 cycles of 94°C for 30 sec, 45°C for 30 sec, 72°C for 2 min; final extension at 72°C for 10 min. For the ND4 locus, primers N4J-8502D (5′-CGT AGG AGG AGC AGC TAT ATT-3′) and N4N-8944D (5′-AAG GCT CAT GTT GAA GCT CC-3′) were used (Fonseca et al. 2001); PCR reactions were similar to COI+II except the final MgCl_2_ concentration was 2.5 mM. The thermal cycler conditions were as follows: 94°C for 2 min; 35 cycles of 94°C for 40 s, 53°C for 40 s, 72°C for 60 s; final extension at 72°C for 10 min. All amplicons were visualized on a 1% agarose gel prior to clean-up and Sanger sequencing as described above. Representative sequences were deposited in GenBank (ND4, accession numbers MZ099906-MZ099910; COI+II, accession numbers MZ099911-MZ099916). Consensus sequences generated here were aligned with other *Argynnis* reference sequences obtained from GenBank using the ClustalW option in MEGA version X (Kumar et al. 2018). Phylogenetic trees were estimated using both neighbor-joining and maximum likelihood methods with a Kimura 2-parameter substitution model and uniform rates among sites, complete deletion of alignment gaps, and 2,000 bootstrap replicates.

## Results

A total of 200 *A. idalia* individuals were sampled between 2016 and 2020; 159 were sampled directly in the field during annual population surveys at FIG-NGTC (82 in 2017, 77 in 2019), and an additional 41 females were sampled as part of a captive rearing and reintroduction project (7–12 per year). In 2019, samples were also collected from sympatric congenerics, *A. cybele* (18 individuals) and *A. aphrodite* (18 individuals), for comparative analysis. Collection locations were recorded for all individuals, but were recoded as ‘west’ (Range 36) and ‘east’ (Ranges 23, B12, C4) for the population genetics analysis due to small sample size for some meadows and some years. The furthest geographical distance between the western and eastern sampling locations is approximately 9.5 km; the three eastern locations are within approx. 2.6 km of each other (as reported in [Keyghobadi et al. 2006]). Previous studies have observed limited dispersal among meadows (Ferster and Vulinec 2010).

Only a single field collected *A. idalia* sample, from 2017, tested positive for a putative *Wolbachia* infection. The MLST profile for this individual was unique and had limited similarity to other *Wolbachia* strains in PubMLST (three out of five loci matching strain types 190, 300, 424, and 522); this positive result was considered spurious and possibly due to surface contamination on the hindleg sample. Three of the *A. idalia* females collected for captive rearing tested positive for *Wolbachia*; MLST profiles for all three were identical and most similar to strain type 43 (four out of five loci plus matching *wsp* allele). *Wolbachia* strain type 43 was isolated from a North American ant species (*Formica occulta*) and is closely related to strains from grass spiders (*Agelenopsis* sp.) and spider wasp (*Evagetes parvus*) (Russell et al. 2009). Offspring for two of the three *A. idalia* females were available for follow-up testing, and none tested positive for *Wolbachia* (data not shown). Given that these females were collected in different years, and the lack of evidence for vertical transmission, these putative infections were considered false positives. In contrast to the *A. idalia* results, all *A. aphrodite* individuals (18 out of 18) and approximately one-third of the *A. cybele* individuals (7 out of 18) tested positive for *Wolbachia*. MLST profiles were generated from a subset of samples (9 from *A. aphrodite*, 5 from *A. cybele*), and all were identified as *Wolbachia* strain type 41, with the additional *wsp* 10 allele.

Nuclear microsatellite alleles were successfully determined for 198 *A. idalia* individuals; three samples were removed from further analysis because their collection locations within FIG-NGTC were ambiguous. The total number of alleles per locus ranged between 10 and 20 (Si13, 10 alleles; Si17, 13 alleles; Si18, 15 alleles; Si31, 20 alleles); the gravid females collected for captive rearing showed equivalent levels of allelic diversity compared to the total sample (Si13, 8 alleles; Si17, 11 alleles; Si18, 11 alleles; Si31, 14 alleles). Twenty-five individuals were genotyped more than once; of these, 309 out of 320 alleles were identical among replicates, suggesting a genotyping error of 3.4%. The final dataset contained 2.6% missing data (1% for Si17, 9.2% for Si18, no missing data for Si13 or Si31). Allelic diversity was similar between the ‘west’ and ‘east’ meadows (Table 1), with no evidence of population differentiation (*F*_*ST*_ = 0.005, *P* = 0.149; *G*_*ST*_ = 0.001, *P* = 0.157); similar results were obtained when the dataset was reanalyzed without Si18 to ensure that missing data were not influencing estimates of population differentiation (*F*_*ST*_ = 0.005, *P* = 0.202; *G*_*ST*_ = 0.001, *P* = 0.203). All loci for both the ‘west’ and ‘east’ groups showed significant deficits in heterozygosity (departure from Hardy-Weinberg equilibrium, *P* < 0.001); the majority of alleles for each locus showed an excess of homozygotes compared to expected values (Supp Fig. 1 [online only]). The frequency of null alleles for each locus estimated under the method of Brookfield (Brookfield 1996) ranged from 12% to 33% (Table 2); similar estimates were obtained using the method of Chakrabotry et al (Chakraborty et al. 1992) (data not shown). Attempts to amplify the *A. idalia* microsatellites in *A. aphrodite* and *A. cybele* were generally unsuccessful, although putative alleles were genotyped for Si18 from both species (data not shown).

**Table 1.** Allelic diversity in the remnant *Argynnis idalia* population in Pennsylvania for four microsatellite loci

		Si13			Si17			Si18			Si31		
	N	A_N_	A_R_	H_O_/H_E_	A_N_	A_R_	H_O_/H_E_	A_N_	A_R_	H_O_/H_E_	A_N_	A_R_	H_O_/H_E_
West (Range 36)	73	10	9.5	0.26/0.77	12	11.6	0.68/0.87	11	10.7	0.29/0.76	18	14.3	0.36/0.91
East (Ranges 23, B12, C4)	122	10	9.3	0.41/0.80	11	10.4	0.62/0.84	14	11.8	0.37/0.76	20	17.3	0.49/0.90

N = number of genotyped individuals, A_N_ = number of alleles, A_R_ = allelic richness, H_O_ = observed heterozygosity, H_E_ = expected heterozygosity.

**Table 2.** Null allele frequencies and 95% confidence intervals for four microsatellite loci calculated under the method of Brookfield (1996)

	Si13	Si17	Si18	Si31
West (Range 36)	28.7 (17.1–39.7)	12.0 (5.0–19.2)	33.2 (21.6–45.3)	31.6 (21.8–41.7)
East (Ranges 23, B12, C4)	32.5 (24.8–40.3)	13.3 (7.7–19.4)	32.2 (23.5–41.4)	31.7 (24.3–39.8)

**Fig. 1.**
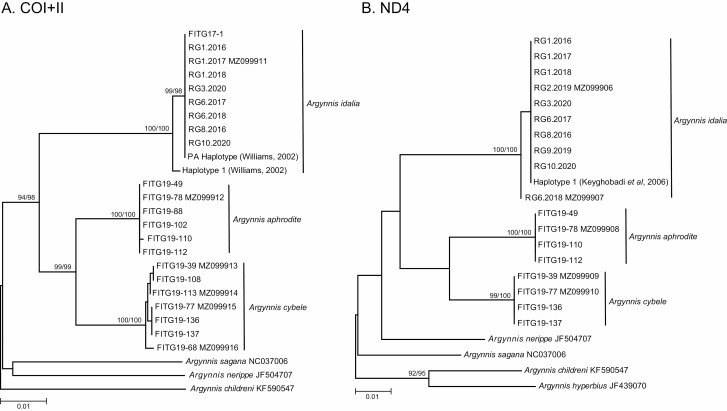
Neighbor-joining phylogenies for mitochondrial loci, COI+II (A, 1,407 base pairs) and ND4 (B, 394 base pairs). Numbers on nodes reflect bootstrap support values greater than 90% (2,000 replicates) for neighbor-joining (left) and maximum likelihood (right) analyses.

Phylogenetic analysis of the mitochondrial COI+II and ND4 loci showed very little intraspecific variation within our *Argynnis* samples (Fig. 1), and both neighbor-joining and maximum likelihood methods produced identical topologies. For the COI+II locus, all *A. idalia* individuals were identical to the PA haplotype identified previously (Williams 2002). All *A. aphrodite* individuals were genetically identical at the COI+II locus except for a single polymorphism in one sample (FITG19-110, Fig. 1A). Higher sequence diversity was found in *A. cybele*, which contained six polymorphisms out of 1407 aligned sites for COI+II. For ND4, the majority of *A. idalia* samples were identical to the previously described H1 haplotype (Keyghobadi et al. 2006); only one sample was identified as H2 (RG6.2018, Fig. 1B), which has a single base difference from H1. All *A. aphrodite* individuals were identical, and *A. cybele* showed a single polymorphism in one sample (FITG19-39, Fig. 1B). For the nuclear 28S ribosomal DNA locus, nine representative *A. idalia* individuals were genetically identical (accession number MZ079856); *A. aphrodite* and *A. cybele* samples were genetically identical to each other (accession numbers MZ079857 and MZ079858) and 1.3% divergent from *A. idalia* (11 variable sites out of 860 aligned positions).

## Discussion

Effective conservation strategies must integrate local and landscape-level environmental variables, such as habitat suitability, with measures of population viability, such as genetic diversity and disease prevalence. In this study, we have focused on the critically imperiled population of eastern regal fritillary, *A. idalia*, in south-central Pennsylvania. Previous studies have shown that the remnant Pennsylvania population is highly divergent from Midwestern and Great Plains populations (Williams 2002, Williams et al. 2003, Keyghobadi et al. 2013), with some evidence of genetic differentiation within the isolated PA population (Keyghobadi et al. 2006). Our analyses of nuclear microsatellite and mitochondrial gene sequence data from *A. idalia* individuals collected over a five year period do not suggest population subdivision among butterflies occupying the western and eastern meadows at FIG-NGTC. However, our ability to accurately detect population structure is complicated by the excess homozygosity observed in our microsatellite data. Deficiencies in heterozygosity and thus departures from Hardy-Weinberg equilibrium in our dataset are likely the result of two main factors: recent demographic history of the FIG-NGTC population and null alleles. Although monitoring over an eight-year period between 1998 and 2005 suggested a relatively stable population density for *A. idalia* at FIG-NGTC (Ferster and Vulinec 2010), large fluctuations have been observed during annual surveys over the past 15 years (Zografou et al. 2017; VT, unpublished data). These recent, potential bottlenecks have likely impacted genetic diversity by reducing the overall population size and by increasing levels of inbreeding among the remaining individuals, leading to excess homozygosity.

Aside from biological factors, the occurrence of null alleles within microsatellite datasets also impacts levels of observed homozygosity. Null alleles have been reported broadly across Lepidopteran studies (Van’t Hof et al. 2007), and commonly result from mutations in primer binding sites or from indel polymorphisms that produce alleles outside of the expected range; unequal amplification of adjacent alleles and stuttering can also result in undetected heterozygosity (Hoffman and Amos 2005, Guichoux et al. 2011). The microsatellite loci utilized here (Williams et al. 2002) likely suffer from several methodological issues leading to null alleles. One locus, Si18, failed to amplify in 18 out of 195 individuals, despite repeated attempts and successful amplification of other loci from the same DNA sample. Three loci (Si13, Si18, Si31) showed high levels of stuttering, which was most disruptive to allele calling for locus Si31 as previously suggested (Keyghobadi et al. 2006); these three loci also contain compound repeats, which can result in cryptic diversity when sequence variants produce amplicons of the same length (Guichoux et al. 2011). In this regard, locus Si17, a simple tetranucleotide repeat, should produce the most reliable results; in our dataset, this locus showed the smallest difference between *H*_*O*_ and *H*_*E*_ (Table 1) and the lowest estimate of null allele frequency (Table 2). To disentangle the biological vs methodological sources of excess homozygosity in the FIG-NGTC population, future studies should employ population genomics approaches, such as genome-wide single-nucleotide polymorphisms (SNPs) panels (Campbell et al. 2019, Dupuis et al. 2020, Trense et al. 2021). Improved genotyping methods are also crucial for captive rearing and reintroduction efforts to ensure adequate genetic diversity in founding populations (Andersen et al. 2014, Daniels et al. 2018).

Unlike their sympatric congeneric *Argynnis* species, the *A. idalia* population at FIG-NGTC does not appear to harbor endosymbiotic *Wolbachia* bacteria. *Wolbachia*-positive individuals were previously identified in Wisconsin populations of *A. idalia* (Hamm et al. 2014), although the specific strain type was not determined. The high prevalence of strain type 41 infection in both *A. aphrodite* (100% of tested individuals) and *A. cybele* (~40% of tested individuals) at FIG-NGTC is concerning, however, given research suggesting horizontal transfer of *Wolbachia* endosymbionts is more successful among closely related hosts (Russell et al. 2009, Zhao et al. 2021), especially those that share food sources (Ahmed et al. 2016), as is commonly observed at FIG-NGTC (data not shown). Monitoring for *Wolbachia* infection status in the FIG-NGTC eastern regal fritillary population will therefore play a crucial role in on-going conservation strategies, including captive rearing and reintroduction efforts, as shown in similar studies (Nice et al. 2009, Fenner et al. 2017, Daniels et al. 2018, Dincă et al. 2018).

Conservation efforts to protect the last remaining eastern regal fritillaries in Pennsylvania have succeeded in allowing several small, isolated subpopulations to persist at FIG-NGTC. On-going captive rearing and reintroduction projects will play a vital role in maintaining *A. idalia* genetic diversity and in monitoring for potential disease. Similar restoration and reintroduction efforts have improved stability in Midwestern and Great Plains populations of *A. idalia* (Huebschman and Bragg 2000, Shepherd and Debinski 2005, Vogel et al. 2007, Shuey et al. 2016); active management strategies will only increase in importance as climate change and other anthropogenic impacts continue to reduce habitat quality and availability for native species.

## Supplementary Data

Supplementary data are available at *Journal of Insect Science* online.

Supplemental Figure 1. Observed and expected number of homozygotes per allele for the four microsatellite loci analyzed in this study. The observed number of homozygotes is shown as a red dot for each allele. Boxplots show expected number of homozygotes calculated via bootstrap analysis in PopGenReport (25th, 50th, 75th percentiles as boxes; 1.5x interquartile range as whiskers).

ieac006_suppl_Supplementary_Figure_1
