# Supplementary material for: Genetic Diversity and Wolbachia (Rickettsiales: Anaplasmataceae) Prevalence Within a Remnant Population of Regal Fritillary, Argynnis idalia (Lepidoptera: Nymphalidae), in South-Central Pennsylvania
Source: J Insect Sci. 2022 Feb 16;22(1):24. doi: 10.1093/jisesa/ieac006 (PMC8849233; doi:10.1093/jisesa/ieac006)
Supplement: ieac006_suppl_Supplementary_Figure_1 [file ieac006_suppl_supplementary_figure_1.pdf]

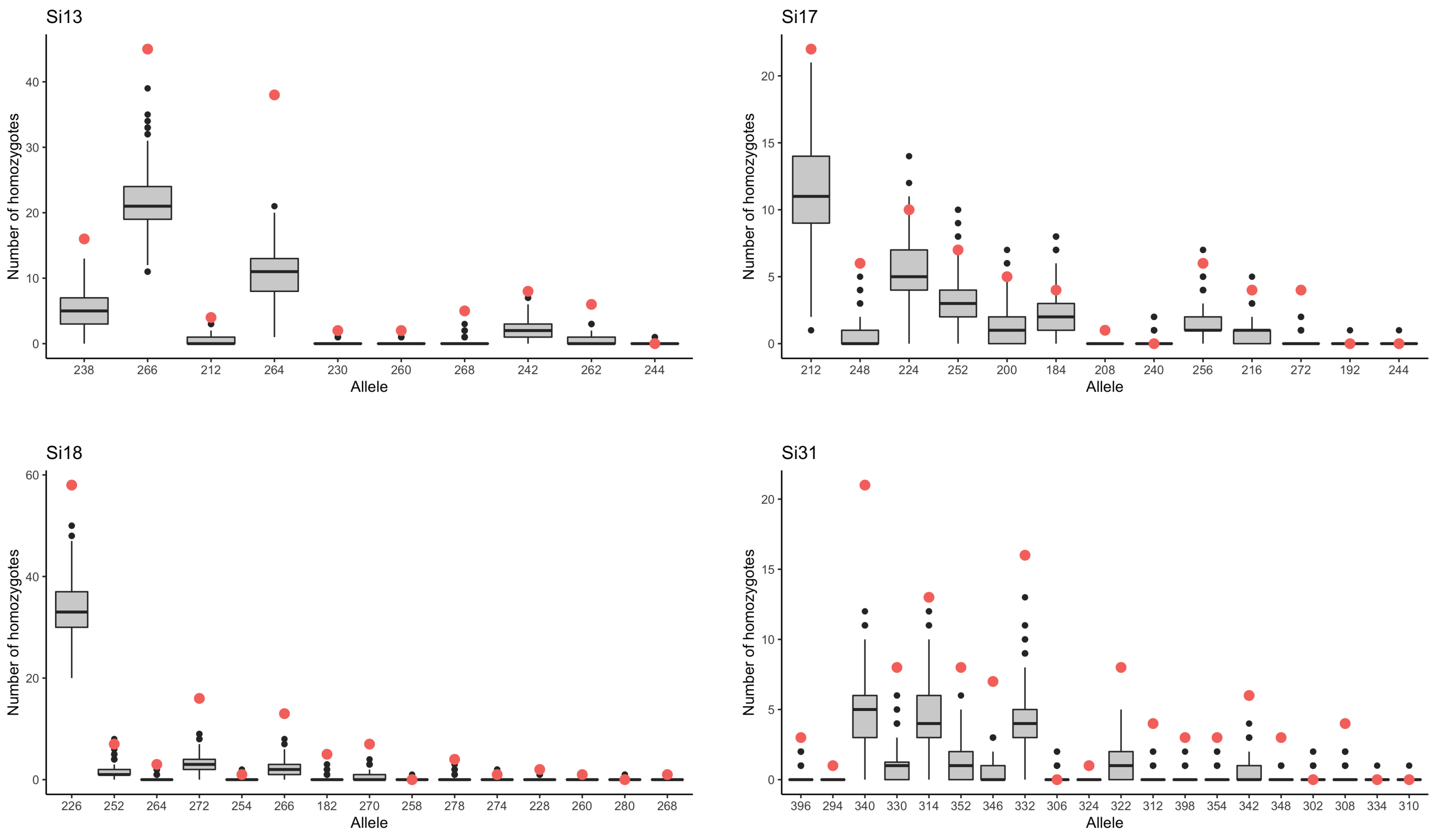

Supplemental Figure 1. Observed and expected number of homozygotes per allele for the four microsatellite loci analyzed in this study. The observed number of homozygotes is shown as a red dot for each allele. Boxplots show expected number of homozygotes calculated via bootstrap analysis in PopGenReport (25th, 50th, 75th percentiles as boxes; 1.5x interquartile range as whiskers).
